# Molecular mechanisms of neurodegeneration in the entorhinal cortex that underlie its selective vulnerability during the pathogenesis of Alzheimer's disease

**DOI:** 10.1242/bio.056796

**Published:** 2021-01-25

**Authors:** Olayemi Joseph Olajide, Marcus E. Suvanto, Clifton Andrew Chapman

**Affiliations:** 1 Division of Neurobiology, Department of Anatomy, University of Ilorin, Ilorin, Nigeria, PMB 1515; 2 Center for Studies in Behavioral Neurobiology, Department of Psychology, Concordia University, Montréal, Québec, Canada H4B 1R6

**Keywords:** Alzheimer's disease, Amyloid beta protein, Apoptosis, Entorhinal cortex, Excitotoxicity, Glia activation, Inflammation, Oxidative stress, Tau

## Abstract

The entorhinal cortex (EC) is a vital component of the medial temporal lobe, and its contributions to cognitive processes and memory formation are supported through its extensive interconnections with the hippocampal formation. During the pathogenesis of Alzheimer's disease (AD), many of the earliest degenerative changes are seen within the EC. Neurodegeneration in the EC and hippocampus during AD has been clearly linked to impairments in memory and cognitive function, and a growing body of evidence indicates that molecular and functional neurodegeneration within the EC may play a primary role in cognitive decline in the early phases of AD. Defining the mechanisms underlying molecular neurodegeneration in the EC is crucial to determining its contributions to the pathogenesis of AD. Surprisingly few studies have focused on understanding the mechanisms of molecular neurodegeneration and selective vulnerability within the EC. However, there have been advancements indicating that early dysregulation of cellular and molecular signaling pathways in the EC involve neurodegenerative cascades including oxidative stress, neuroinflammation, glia activation, stress kinases activation, and neuronal loss. Dysfunction within the EC can impact the function of the hippocampus, which relies on entorhinal inputs, and further degeneration within the hippocampus can compound this effect, leading to severe cognitive disruption. This review assesses the molecular and cellular mechanisms underlying early degeneration in the EC during AD. These mechanisms may underlie the selective vulnerability of neuronal subpopulations in this brain region to the disease development and contribute both directly and indirectly to cognitive loss.

This paper has an associated Future Leader to Watch interview with the first author of the article.

## Abstract (precis)

**Summary:** This review evaluated the molecular mechanisms that may explain the vulnerability and susceptibility of the entorhinal cortex to early neurodegeneration during the pathogenesis of Alzheimer's disease.

## Introduction

Alzheimer's disease (AD) is a progressive neurodegenerative disorder that results in widespread neuropathology throughout the brain, and severe disruption of cognitive and mnemonic function (Calderon-Garcidueñas and Duyckaerts, 2018; Jahn, 2013). AD involves the development of extracellular plaques composed of insoluble amyloid beta protein (Aβ), and intracellular tangles of hyperphosphorylated tau protein (Braak and Braak, 1991, 1995; Desikan et al., 2012; Lue et al., 1999). In the latter stages of AD, as symptoms worsen, neurodegeneration is widespread throughout the cortex, and medial temporal lobe structures including the hippocampal formation are profoundly affected (Calderon-Garcidueñas and Duyckaerts, 2018; Guo et al., 2020; van Hoesen et al., 1991). Dysfunction of the entorhinal cortex (EC) and hippocampus is thought to mediate much of the cognitive impairment observed early in the progression of AD. The early stages of cognitive decline in AD can be relatively mild and difficult to distinguish from cognitive declines in normal aging and other forms of dementia, and typically involve deficits in memory or other cognitive functions, and increased depression (Anderson, 2019). The hippocampus and surrounding parahippocampal cortical regions are well known to be critical for declarative memory formation (Lee et al., 2020), and the EC is thought to be central for memory and spatial functions of the hippocampus (Sugar and Moser, 2019).

A substantial body of research has linked dysfunction of the EC to cognitive impairments experienced early in the progression of AD, and there is also a growing understanding of the factors that may make the EC particularly susceptible to molecular degeneration (Criscuolo et al., 2015, 2017; Guo et al., 2020; Khan et al., 2014; Scharfman and Chao, 2013; Stranahan and Mattson, 2010; Terni et al., 2010; Yang et al., 2018). One factor may be neurotrophic support, and the vulnerability of EC to AD-type neurodegeneration has been attributed to the dysregulation of brain-derived neurotrophic factor (BDNF) and the ARMS/Kidins220 scaffold protein, both of which are critical for neurotrophic support to the EC (Criscuolo et al., 2015; Scharfman and Chao, 2013). Selective degeneration of synapses in entorhinal neurons due to activation of stress-related kinases and neuroinflammatory processes involving activation of microglia receptor for advanced glycation end products (RAGE) may also contribute to the vulnerability of the EC in the early stages of amyloid-dependent neurodegeneration in mice (Criscuolo et al., 2017; Fang et al., 2010). Further, neurons in the EC layer II appear to undergo particularly marked AD-related molecular degeneration that involves downregulation of N-methyl-D-aspartate (NMDA) subtype 1 glutamate receptor, muscarinic acetylcholine receptor 1, and γ-Aminobutyric acid type A (GABAA) receptor delta (Liang et al., 2007; Stranahan and Mattson, 2010).

The development and advancement of neuropathogenesis in most neurodegenerative diseases is governed both by the selective initial vulnerability of particular neuronal subpopulations to the disease pathology, and a generally predictable pattern of gradual spread of pathology across multiple brain regions (Fu et al., 2018). Multiple studies in human subjects and experimental models have proven convincingly that the first signs of AD-related atrophy occur in the EC. An important component of the pathology is the accumulation of Aβ in the outer EC layers, particularly in layer II, which is the major source of excitatory input into the hippocampus where it is believed to incite neurotoxic cascades that ultimately cause profound neuronal death (Gómez-Isla et al., 1996; Götz et al., 2009; Kordower et al., 2001; Price et al., 2001; Stranahan and Mattson, 2010; van Hoesen et al., 1991). Substantial progress has been made in understanding the pathogenesis of AD (Chen and Mobley, 2019; Guo et al., 2020; Sanabria-Castro et al., 2017). However, the molecular characteristics of EC neurons that underlie their selective vulnerability to Aβ and other triggers of AD pathology early during the pathogenesis of AD have yet to be characterized extensively. For example, it is not clear how proteins such as Aβ, that are ubiquitously expressed, accumulate and spread selectively in vulnerable neurons, but not in neighboring cell types and brain regions. Understanding the basis of this selective neuronal or regional vulnerability is key to gaining insights into the molecular underpinnings of neurodegenerative diseases like AD, and could aid the development of potent therapeutic strategies. In this review, we analyzed emerging evidence on the molecular and cellular mechanisms that may account for the selective vulnerability of neuronal subpopulations in the EC early in AD.

## The entorhinal cortex in early stages of AD

Early declines in memory function are associated with the onset of neuronal and synaptic degeneration in the EC, hippocampus and posterior cingulate cortex (Fennema-Notestine et al., 2009; Gómez-Isla et al., 1996; Khan et al., 2014; Selkoe, 2002; Stranahan and Mattson, 2010). The EC appears to be particularly susceptible to early damage, and neurofirillary changes are first observed in the transentorhinal region (Braak and Braak, 1991, 1998). The EC is also disrupted during mild cognitive impairment, which often precedes AD, in which there is a thinning of rhinal cortices and hippocampal atrophy (Whitwell et al., 2007; Zhou et al., 2016). Reductions in the volume of the EC can help predict whether preclinical cognitive impairments will progress into AD (De Toledo-Morrell et al., 2000), and the volume of the anterolateral EC is also associated with early cognitive decline in patients who develop AD (Olsen et al., 2017).

A crucial objective in Alzheimer's research is the identification of biomarkers that can promote early diagnosis and treatment of the disease. The belief is that pre-symptomatic intervention will be far more potent than applying therapy after the onset of disease. The progressive pathological changes that occur in the EC during early AD can serve as effective biomarkers that may aid prediction and diagnosis of AD long before its clinical manifestation (Márquez and Yassa, 2019; Leandrou et al., 2020; Zhou et al., 2016). *In vivo* quantification of atrophy and molecular changes in the EC could also improve monitoring and evaluation of the effectiveness of therapeutic strategies. Holbrook et al. (2020) recently found that thinning of the anterolateral EC occurs very early in AD and correlates with upregulated amyloid and tau in cerebrospinal fluid. The atrophy in the anterolateral EC was more strongly associated with cognitive decline in patients as compared to thinning in the posteromedial EC, leading to the suggestion that the anterolateral EC can serve as an effective anatomical biomarker for early detection of AD. Similarly, the use of texture analysis, a quantitative method that can detect smaller-scale neurodegenerative changes, has found that alterations in the EC predicted AD development more readily than hippocampal atrophy (Leandrou et al., 2020), thereby buttressing the use of degeneration in the EC as an important biomarker for AD.

**Fig. 1.**
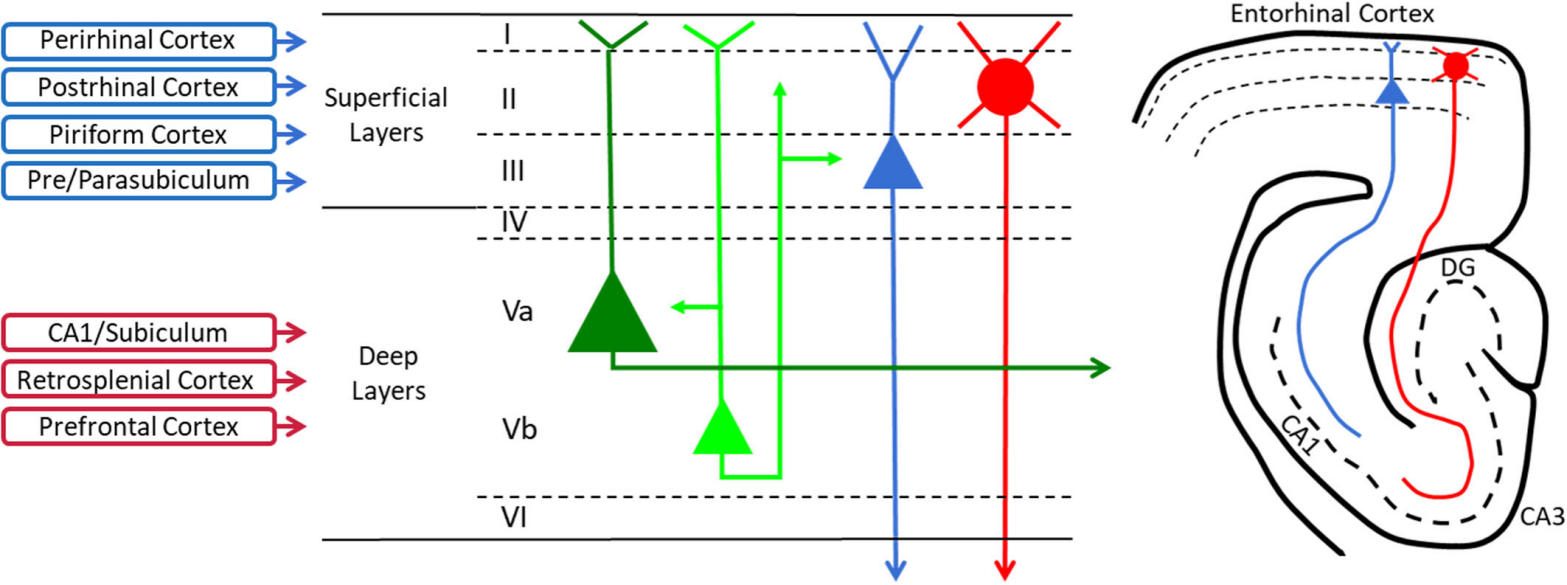
**A simplified schematic diagram outlines the major cortical inputs to the entorhinal cortex, the principle neuronal subtypes within each layer, and the major outputs to the hippocampal formation.** The superficial entorhinal cortex receives major inputs from the postrhinal, perirhinal, and piriform cortex and the deep layers receive inputs from the CA1 of the hippocampus, the subiculum, retroslpenial cortex, and prefrontal cortical areas. Major cortical inputs terminate in superficial layers I-III and terminate on stellate neurons in layer II and pyramidal neurons of layer III. Layer II stellate neurons project heavily to the dentate gyrus (DG) and CA3 area via the performant path, and Layer III neurons form the temporoammonic input to the CA1 region. Outputs of the hippocampus from the CA1 region and subiculum project primarily onto deep layer neurons in layer V, and deep layer neurons project widely to neocortical areas. Adapted from Nilssen et al. (2019) and Witter et al. (2017).

The EC provides the hippocampus with most of its cortical sensory and associational input, and degradation within the EC can therefore disrupt entorhinal function, as well as hippocampal processing that is dependent on entorhinal inputs (Fig. 1). The superficial layers of the EC receive widespread inputs from cortical regions via the perirhinal, postrhinal and piriform cortices, and layer II and III EC neurons in turn provide the main source of cortical sensory input to the hippocampus (Burwell, 2000; Witter et al., 2017). EC layer II stellate neurons, which are profoundly affected in AD (Stranahan and Mattson, 2010), are the origin of the perforant path input to the dentate gyrus (DG) and hippocampal CA3 region. Layer III EC pyramidal neurons project to the CA1 region via the temporoammonic pathway. The early memory loss in AD is thought to be related to the progressive dysfunction and deterioration of the EC and the targets of these pathways in the DG and CA3 and CA1 areas in the hippocampus (Llorens-Martín et al., 2014). Outputs from the CA1 and subiculum project to pyramidal neurons in the deep layers of the EC, and deep layer neurons project to both the superficial layers and much of the neocortex (Burwell, 2000; Witter et al., 2017). Thus, neurodegeneration within the EC may affect cognitive and mnemonic function in AD through impacts on both hippocampal and neocortical function.

Deterioration within the medial EC (MEC) versus lateral EC (LEC) may contribute in different ways to the symptomatology of AD (Nilssen et al., 2019; Schröder et al., 2015). The MEC has an important role in spatial processing and navigation (Sugar and Moser, 2019) and is linked functionally with the parahippocampal cortex and posteromedial cortical regions (Burwell, 2000; Schröder et al., 2015). The LEC is more strongly linked to object identification and olfactory processing, and receives inputs from the perirhinal cortex and anterior cortical regions (Deshmukh and Knierim, 2011; Schröder et al., 2015; Young et al., 1997). The LEC also appears to be affected earlier and more severely in AD than the MEC; the LEC shows earlier deposition of plaques and neurofibrillary tangles in preclinical AD and pathology spreads from LEC to parietal cortex (Braak and Braak, 1995; Braak et al., 2006; Khan et al., 2014). Early deterioration in the LEC may therefore contribute to early cognitive deficits in AD, and because the hippocampus relies upon the EC for sensory and associational inputs, early dysfunction within the EC may disrupt cognitive functions by impairing the nature of hippocampal inputs.

Although the origin of AD-related degenerative changes has been extensively linked to regions of the EC, subcortical structures are also early targets of AD, and degeneration in subcortical regions may contribute to cortical degeneration during AD. For example, selective vulnerability to early AD has been observed in neurons of the nucleus basalis of Meynert (NbM), the largest of the cholinergic cell clusters constituting the basal forebrain that widely innervate the neocortex (Braak and Del Tredici, 2015; Hanna Al-Shaikh et al., 2020). Extensive loss of grey matter in the NbM that preceded degeneration in the EC was also reported in subjects exhibiting elevated levels of Aβ in the cerebrospinal fluid (Schmitz and Spreng, 2016). In addition, baseline NbM volumes were found to predict future rates of structural degeneration in the EC and perirhinal cortices (Fernández-Cabello et al., 2020), raising the possibility that degeneration in the NbM may contribute to EC pathology during the development of AD. Similarly, Arendt et al. (2015) reported that early-stage neuronal death in the EC of AD patients correlates with loss of neurons in the locus coeruleus and NbM, further indicating multiple brain regions are susceptible to early degeneration in AD. Additional research from animal models, however, including critical pathophysiological and molecular analysis to correlate with changes in the entorhinal region, will be needed in order to advance the hypothesis that degeneration in the EC is driven by earlier changes in subcortical regions. The hypothesis that AD originates in the EC and spreads to other cortical and subcortical structures has been supported in both humans and rodents, and continues to be the prevailing model of pathological progression in AD.

## Selective vulnerability to the effects of amyloid-β protein on NMDA glutamate receptors

Aβ accumulation is a major driver of molecular events in AD that lead to synaptic and neuronal dysfunction, through mechanisms including increases in glutamate release from neurons and glia, overactivation of NMDA glutamate receptors (NMDARs), excitotoxicity, and cell death (Parameshwaran et al., 2008; Tackenberg et al., 2013). NMDARs are activated by both presynaptic glutamate release and postsynaptic depolarization, and strong synaptic activation normally contributes to processes of learning and memory by inducing lasting increases in the strength of glutamatergic synaptic transmission (Findley et al., 2019; Rudy et al., 2015). Early in the pathophysiology of AD however, increases in soluble Aβ contribute to aberrant glutamate release that facilitates excitatory synaptic transmission and enhances activation of NMDARs (Findley et al., 2019; Kamenetz et al., 2003; Li and Tsien, 2009; Rudy et al., 2015). The excessive release of glutamate that is caused by Aβ could further result in the inhibition of axonal transport and eventual neurodegeneration (Hiruma et al., 2003). In addition, excessive activation of NMDARs by Aβ protein may contribute to the early neurodegeneration that is observed in the EC during AD. The idea that NMDARs contribute to the early pathogenesis of AD in the EC is supported by findings that bilateral microinjection of either Aβ or NMDA in the rat EC results in selective cognitive deficits resembling those observed in early phases of AD, and also results in the development of reactive astroglia response and plaque-like deposition (Sipos et al., 2007). We have also recently found that exposing EC slices to Aβ_1-42_ rapidly enhances excitatory postsynaptic potentials in layer II through activation of NMDARs (Suvanto and Chapman, unpublished observations). Exaggerated synaptic strengthening contributes to increased activation of the hippocampus early in the progression of AD during mild cognitive impairment (Dickerson et al., 2005; Putcha et al., 2011), and the increase in excitatory transmission occurs prior to neuronal degeneration, and is central to disease progression (Findley et al., 2019; Parameshwaran et al., 2008; Rudy et al., 2015; Zhang et al., 2016). The rapid NMDA receptor dependent facilitation of excitatory transmission in the EC is therefore likely to both interfere with neural processes of learning and memory, and to contribute to increases in hyperexcitability and excitotoxicity leading to neuronal degeneration.

The EC may be particularly vulnerable to excessive NMDA receptor mediated glutamatergic signaling because, in addition to postsynaptic receptors, the EC also contains NMDARs on presynaptic terminals that increase calcium (Ca^2+^)-dependent glutamate release (Berretta and Jones, 1996; Lench et al., 2015; Woodhall et al., 2001), and the activation of NMDARs by Aβ can increase excitotoxicity by elevating synaptic release of glutamate (Rudy et al., 2015). Presynaptic NMDARs in the EC, which are present in both superficial and deep layers, are tonically active and facilitate the release of glutamate onto principal neurons (Lench et al., 2014; Woodhall et al., 2001). Aβ activation of presynaptic terminals, which contain NR2B subunits, may therefore strongly enhance excitability by increasing release of glutamate. In addition, soluble Aβ can contribute to increased glutamate availability through activation of astrocytic α-7 nicotinic receptors located on astrocytes, which then elevate astrocytic Ca^2+^ and increase release of astrocytic glutamate (Findley et al., 2019; Rudy et al., 2015). Aβ may also interfere with astrocytic mechanisms responsible for clearing excess glutamate from the synapse (Rudy et al., 2015), and the combination of factors may substantially elevate glutamate-mediated excitotoxicity in the EC.

The selective vulnerability of the EC to early degeneration during AD may also be related to the susceptibility of NMDARs genes to genetic variations such as single nucleotide polymorphisms (SNP), which have been associated with early onset AD (Lee et al., 2017). For example, rs1806201, which is within exon 13 of the NR2B gene locus, may play a role in modulating susceptibility to AD (Andreoli et al., 2014), and the frequency of the Ht2-AG haplotype in the NR3A gene may also be higher in AD patients, indicating that NR3A variants may increase the risk of AD (Liu et al., 2009). Similarly, mRNA and protein levels of NMDARs subunits are altered in the EC during AD (Hynd et al., 2004b; Sze et al., 2001). We have also recently found that application of Aβ_1-42_ to EC slices from wild-type rats induces a reduction in the presynaptic protein synaptophysin, the postsynaptic scaffolding protein PSD-95, as well as marked reductions in mRNA expression of NMDAR subunits NR2A and NR2B (Olajide and Chapman, unpublished findings). These findings are similar to those in the hippocampus which also show downregulation of NR2A and NR2B mRNA expression in AD (Hynd et al., 2004a; Jacob et al., 2007; Mishizen-Eberz et al., 2004). In addition, Aβ-mediated reduction in glutamate receptors and rapid degeneration of synaptic elements in hippocampal neurons were attributed to activation of apoptotic factors (Snyder et al., 2005; Liu et al., 2010). This further emphasizes the importance of NMDARs in activating both presynaptic and postsynaptic mechanisms that mediate aberrant synaptic strengthening in the EC early in the pathogenesis of AD. However, the intracellular signaling pathways that mediate these changes, and the features of EC neurons that make them vulnerable to these aberrant increases in synaptic transmission still need to be determined.

Excessive glutamatergic signaling and activation of NMDA receptors contributes directly to Ca^2+^-mediated excitotoxicity and neuron death (Arundine and Tymianski, 2003; Berdichevsky et al., 1983; Choi et al., 1987; Choi, 1988; Hynd et al., 2004a; Tymianski and Tator, 1996). NMDARs are Ca^2+^-permeable, and the influx of Ca^2+^ can trigger increases in the phosphorylation of α-amino-3-hydroxy-5-methyl-4-isoxazolepropionic acid (AMPA) glutamate receptors, their recruitment to the postsynaptic membrane, and growth of dendritic spines (Palop and Mucke, 2010). In AD, excess glutamate release induced by soluble Aβ results in abnormal elevations in postsynaptic Ca^2+^ that contribute to lasting increases in AMPA receptor mediated synaptic transmission (Palop and Mucke, 2010; Selkoe, 2002; Spires-Jones and Hyman, 2014). In addition, excessive influx of Ca^2+^ and release of Ca^2+^ from intracellular compartments can overwhelm Ca^2+^-regulatory mechanisms and lead to cell death (Wang and Reddy, 2017; Zhang et al., 2016). Activation of extrasynaptic NMDARs that predominantly contain the NR2B subunit is a central mechanism of Ca^2+^-dependent neurotoxicity (Butterfield and Pocernich, 2003; Danysz and Parsons, 2012; Liu et al., 2019; Texidó et al., 2011; Wang and Reddy, 2017; Zhang et al., 2016). Late in the progression of the neuropathology of AD, the activation of NMDA receptors by elevated levels of Aβ leads to synaptic and neuronal degeneration through Ca^2+^-dependent excitotoxicity (Palop and Mucke, 2010) and cell death through oxidative stress and deterioration of synapses and neurons (Perry et al., 2002; Berridge, 2011; Rudy et al., 2015).

## Drivers of molecular neurodegeneration and vulnerability of the EC during AD

Advances in molecular neuroscience techniques are beginning to spotlight mechanisms of neurodegeneration during the pathogenesis of AD through analysis of the neurogenomic properties of vulnerable neurons in key brain areas like the EC. Identifying the specific genes associated with the vulnerability of the EC to AD provides a powerful way to assess the biochemical characteristics of neurons in the EC that increase their propensity to accumulate disease-related molecular and cellular changes. Liang et al. (2007) combined laser capture microdissection and analysis of gene expression using microarrays to characterize EC neurons and disease-related changes in gene expression in AD. When compared to other brain areas that are less vulnerable to the histopathological and metabolic features of AD, the EC and hippocampus had the highest numbers of genes that showed significantly altered expression (43 and 37, respectively). In particular, the EC was found to have profoundly upregulated glycogen synthase kinase 3 beta (GSK3β), and calcium/calmodulin-dependent protein kinase II delta (CaMK2D; Liang et al., 2007). The GSK3 hypothesis of AD (Crews and Masliah, 2010; Hooper et al., 2008) postulates that GSK3 hyperactivity mediates many of the pathological hallmarks of AD, including increased production and accumulation of Aβ, neuroinflammatory responses, cholinergic deregulation, tau hyperphosphorylation, apoptotic proteins dysfunction, and ultimately memory impairment (Embi et al., 1980; Giese, 2009; Hoshi et al., 1996; Hoshi et al., 1996; Lucas et al., 2001; Turenne and Price, 2001). Similarly, the deregulation of CaMK2, a major postsynaptic protein at excitatory synapses, is thought to contribute to Ca^2+^-dependent toxicity that results in synaptic degeneration and neuronal loss (Ghosh and Giese, 2015; Sabbir, 2018; Ly and Song, 2011). In addition, in comparison to other brain regions, the EC shows the lowest expression of nuclear transcription factor Y subunit beta (NFYB; Liang et al., 2007). Alteration of transcriptional regulation factor NFYB and other related transcription genes have also been demonstrated to mediate molecular neurodegeneration and apoptotic processes (Chen et al., 2013; Ly et al., 2013). Perturbations in the genetic expression of these pathogenic markers in the EC during the early phase of AD, as found by Liang and co-workers (Liang et al., 2007), may therefore be an important underpinning of the EC's selective vulnerability to development of the disease.

A more recent study used single-nucleus RNA-sequencing (SnRNA-seq) to generate large-scale transcriptomic profiles of individual EC cells from control and post-mortem AD brains, in order to assess cell-type-specific differences in gene expression patterns that may influence AD susceptibility; subpopulations of cells with unique networks of co-regulated genes and functions were identified across different cell types in the EC of AD brains (Grubman et al., 2019). Importantly, it was found that although the AD risk gene apolipoprotein E (APOE) was repressed in astrocyte subpopulations and in AD-related oligodendrocyte progenitor cells, it was upregulated in an AD-specific microglial subpopulations within the EC, suggesting that these specialized cells contribute to disease susceptibility of the EC during AD. A selectively vulnerable subpopulation of excitatory neurons in the EC, which shows a corresponding selective depletion as the disease progresses, has also been identified using SnRNA-seq in post-mortem AD brains (Leng et al., 2020, preprint). Interestingly, this study identified nuclear receptor RAR-related orphan receptor beta (RORB), which is a developmental driver of neuronal subtype identity (Jabaudon et al., 2012; Oishi et al., 2016), as a marker for the selective vulnerability of excitatory EC neurons. In addition, there was a downregulation of genes involved in homeostatic function in reactive astrocytes, which are a subpopulation of glia cells in the EC. Surprisingly, subpopulations of inhibitory neurons in the EC showed no differences in vulnerability, which contrasts with the findings of Petrache et al., (2019) that have shown that parvalbumin-positive interneurons show early dysfunction in the EC that contributes to hyperexcitability in the EC during very early stages of AD pathology. Studies of genomic profiles of particular glia and neuronal subtypes within the EC, and of the intermediate mechanisms that impact cell function, will continue to clarify the mechanisms of AD progression in the EC.

## Loss of modulatory transmitters in the EC

A substantial contributor to cognitive decline in AD is thought to be the deterioration of neuromodulatory transmitters including noradrenergic, serotonergic, and cholinergic neurons (Hampel et al., 2018; Hardy et al., 1985; Trillo et al., 2013). Neuronal processes in the hippocampus and EC that contribute to sensory processing and memory encoding are known to be dependent on cholinergic transmission, and the deterioration of cholinergic neurons in the basal forebrain is thought to be a strong contributor to early memory dysfunction (Francis et al., 1999; Hampel et al., 2018). Cholinergic inputs play a central role in the generation of theta- and gamma-frequency EEG activities by depolarizing neurons and promoting mechanisms of neuronal synchronization during memory encoding (Dannenberg et al., 2016; Dickson et al., 2000). Reduced theta and gamma frequency activity in the EC and hippocampus likely contributes to cognitive decline by disrupting rhythmic population activity contributing to memory processing (Colom et al., 2010; Nakazono et al., 2018; Pena-Ortega et al., 2012). In addition, acetylcholine is known to inhibit excitatory synaptic transmission in the EC by reducing glutamate release (Dannenberg et al., 2016; Hamam et al., 2007). Loss of cholinergic inputs during AD may therefore contribute to excitotoxicity by enhancing glutamate release. Similarly, serotonin hyperpolarizes neurons and suppresses excitatory synaptic transmission in the EC (Carter and Chapman, 2019; Ma et al., 2007), and loss of serotonergic inputs may also facilitate synaptic transmission during the progression of AD.

In addition to increases in excitatory synaptic transmission, disruption of inhibitory synaptic transmission in the LEC is thought to contribute to excitotoxicity (Xu et al., 2020). In a knock-in mouse model of AD expressing a mutant form of human Aβ precursor protein, cells in LEC show loss of parvalbumin containing interneurons that regulate excitability, leading to hyperexcitability of principle neuron and increases in EPSPs, and the disruption of parvalbumin neurons was observed earliest in the LEC (Petrache et al., 2019). The EC is a key region in epileptogenesis (Vismer et al., 2015), and the dysfunction of inhibitory neurons in the EC during the progression of AD may promote burst discharges that may further promote excitotoxicity, and contribute to the early susceptibility of this brain area to AD-related changes.

## Early EC degeneration during AD may be mediated by mitochondrial dysfunction and oxidative stress

The production of reactive oxygen species (ROS) at physiological levels in mitochondria during aerobic respiration has a positive role in cellular signaling, but if ROS exceed the cellular antioxidant capacity, ROS can disrupt the redox balance and result in oxidative stress. Oxidative stress alters the structure of macromolecules through the formation of cross-linkages, and leads to the functional alteration of proteins, lipids, and nucleic acids. Effects on DNA and RNA are a major trigger for neuronal degeneration, and several lines of evidence including our previous studies suggest that oxidative stress resulting from mitochondrial dysfunction are at the forefront of neuronal death during AD pathogenesis (Abolhassani et al., 2017; Albrekkan and Kelly-Worden, 2013; Butterfield and Halliwell, 2019; Butterfield et al., 2010; Cavallucci et al., 2013; Moreira et al., 2010; Mourier, 2016; Olajide et al., 2017, 2018; Petrozzi et al., 2007; Tramutola et al., 2017). In AD, Aβ can accumulate in mitochondria where it impairs mitochondrial dynamics and upregulates oxidative stress by impairing mitochondrial respiratory function and the production of adenosine triphosphate (ATP). The increased production of ROS induced by Aβ peptides can then cause the release of cytochrome c and apoptosis-inducing factor, thereby driving neuronal apoptosis (Moreira et al., 2010).

Oxidative stress and mitochondria dysregulation appears to be an important early molecular driver of AD-type neuropathology within the EC. Oxidative stress manifests very early during AD, leading to the hypothesis that it then drives further pathogenesis mediated by Aβ (Wang et al., 2014). Neuronal populations are differentially sensitive to the toxicity of oxidative stress and show earlier functional and structural degeneration during the pathogenesis of degenerative diseases (Wang and Michaelis, 2010). Hippocampal CA1 and CA2 pyramidal neurons respond differently to oxidative stress despite their proximity and morphological similarity, and agents that induce oxidative stress cause widespread cell death in the CA1 region, while CA3 neurons are mostly spared (Sarnowska, 2002; Vornov et al., 1998; Wang et al., 2005). EC neurons are also particularly susceptible to damage by oxidative stress and mitochondria dysfunction. Using proteomic analysis, Terni et al. (2010) examined whether proteins are locally modified by oxidative stress at the first stages of AD-related pathology when neurofibrillary pathology is restricted to the entorhinal and transentorhinal cortices (stages I/II of Braak). They found that oxidative damage was present during the first, clinically silent, stage of AD-related pathology, and that the α subunit of the mitochondrial ATP-synthase is distinctly lipoxidized in the EC during Braak stages I/II compared with age-matched controls. Furthermore, although the electron transport chain expressed by the mitochondrial complex I activity was not affected, ATP-synthase activity was found to be significantly lower in Braak stages I/II than in age-matched controls.

Oxidative damage to RNA in subjects with AD has been assessed using immunoreactivity for the oxidised nucleoside 8-hydroxyguanosine (8OHG), and it is elevated in neuronal cytoplasm of brain regions that are vulnerable to AD pathology including the EC, hippocampus, subiculum and temporal neocortex (Nunomura et al., 2001). Levels of 8OHG were highest early in the disease long before histopathological changes were detected, and this supports the idea that the vulnerability of neurons in the EC and other brain structures to early degeneration during AD may be related to its susceptibility to damage by oxidative stress. Furthermore, oxidative stress in the EC may also contribute to the expression of clusterin, which is a secreted glycoprotein. Plasma levels of clusterin are associated with early atrophy in the EC and the clinical progression of AD (Thambisetty et al., 2010), and clusterin has been identified as a genetic determinant of AD in a genome-wide study (Lambert et al., 2009). Interestingly, oxidative stress induced with hydrogen peroxide in human diploid fibroblasts has been shown to increase expression of clusterin as reflected in increased mRNA levels (Chen et al., 2001). This further suggests that oxidative stress plays an important role in triggering pathological mechanisms early in the progression of AD.

Collectively, these studies provide compelling argument for the hypothesis that oxidative stress and mitochondria dysfunction may drive early degeneration in the EC during AD and other neurodegenerative pathologies. The importance of oxidative stress as a molecular driver of degenerative mechanisms in EC neurons, and the early occurrence of oxidative stress in the EC, suggests that it is an important component of the selective vulnerability of the EC. Major hypotheses of the role of oxidative stress in AD propose that ROS dysregulation precedes, and may in fact induce, the extracellular accumulation of Aβ in neurons. However, both degenerative processes may occur simultaneously and with a certain degree of co-dependence as suggested by observations that oxidative stress occurs predominantly in Aβ_1-42_-rich areas like the hippocampus as compared to Aβ_1-42_-poor brain regions (Hensley et al., 1995; Butterfield and Boyd-Kimball, 2018). The EC is one of the earliest temporal lobe structures to show both oxidative impairment and Aβ accumulation, and it is likely that an interplay between these two factors may underlie the susceptibility of this brain area to AD-related degeneration.

## Possible roles of neuroinflammation and glia activation in selective vulnerability of the EC

**Fig. 2.**
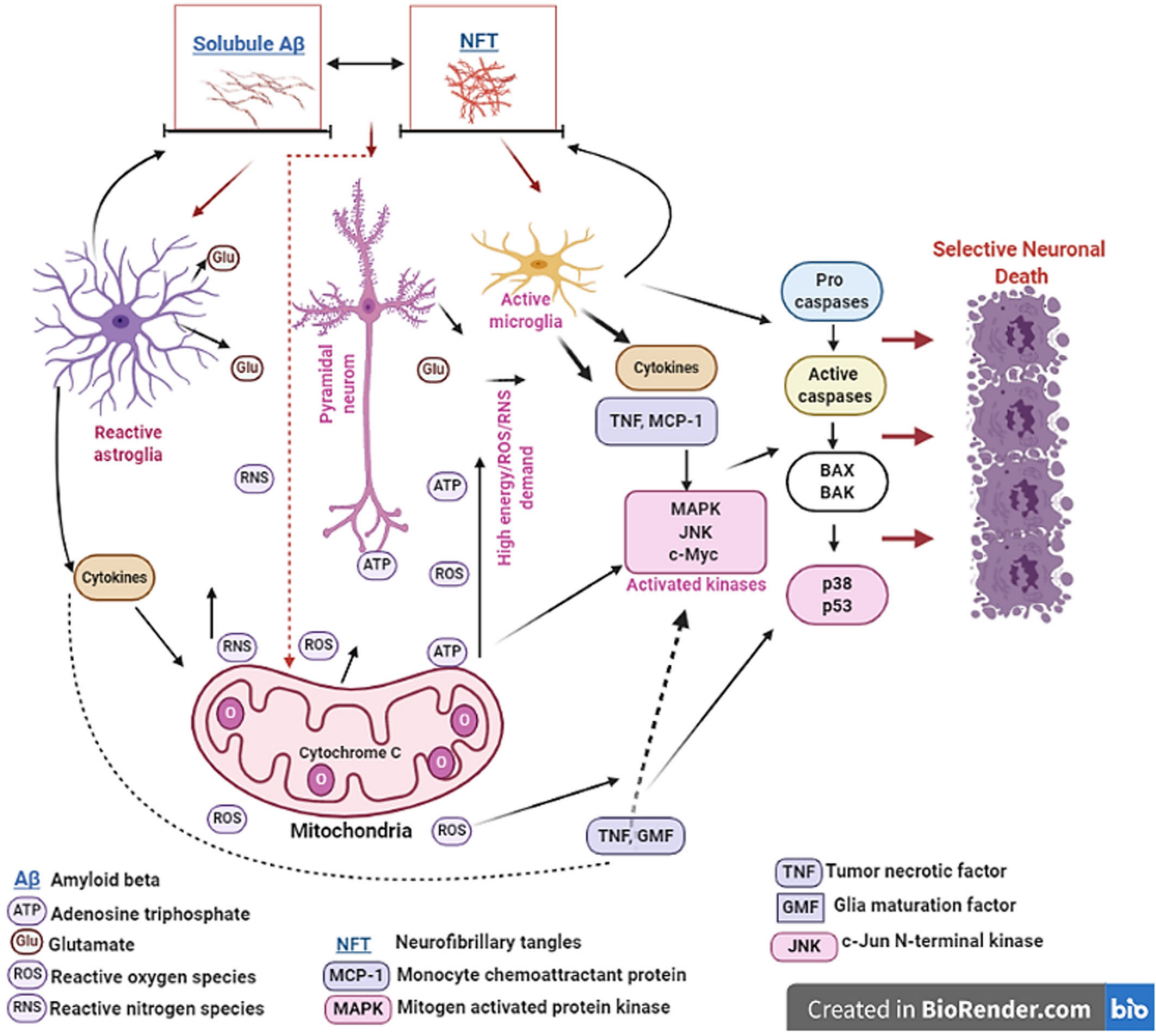
**Schematic diagram of cellular and molecular factors that may contribute to the selective vulnerability of the entorhinal cortex (EC) during Alzheimer's disease (AD).** Neural cells in the EC are the first to show signs of AD-related neuropathology, a phenomenon that may be related to its unique intrinsic cellular and molecular composition. Soluble amyloid beta (Aβ) and neurofibrillary tangles (NFT) may induce neurotoxicity in the EC by perturbing signaling molecules in neurons and by activating neuroglia (astroglia and microglia). One distinct characteristic of cells in the EC is a propensity for relatively high energy demand, which is supplied by the mitochondria in the form of ATP (adenosine triphosphate) and other signaling molecules like reactive oxygen species/reactive nitrogen species (ROS/RNS). However, due to the duality of the highly reactive species ROS/RNS, the high demand for these molecules, even subtle neurotoxic effects of Aβ and NFT can lead to intrinsically high oxidative stress in EC neurons, making them selectively vulnerable. High oxidative stress in neurons may be coupled to production of other neurotoxic molecules like cytokines from neighboring glia cells, thereby increasing overall stress responses and susceptibility of neural subpopulation in the EC to degeneration. Vulnerable neurons like those in the EC are also characterized by mitochondrial dysfunction, possibly due to high oxidative state in these cells, making the mitochondria vulnerable to further dysregulation by the direct toxic action of Aβ and NFT. Furthermore, the EC is highly rich in glutamate containing cells, and Aβ and NFT can stimulate excessive release of glutamate from both neurons and astroglia, thus increasing the susceptibility of this brain area to excitotoxic stimuli. Excessive release of highly reactive oxygen/nitrogen species and glutamate can exacerbate inflammatory responses in neuroglia and cause further release of pro-inflammatory molecules and cell death kinases including tumor necrotic factor (TNF), monocyte chemoattractant protein (MCP-1), mitogen activated protein kinase (MAPK), glia maturation factor (GMF), c-Jun N-terminal kinase (JNK) and c-Myc, factors that are known to underlie early EC degeneration during AD. When combined with high DNA oxidation, activation of these kinases can lead to dysregulation of cell cycle regulatory proteins including p53 and Bax/Bak which have also been suggested to be easily triggered in vulnerable neurons, and to cause selective neuronal death. The arrows link these mechanistic factors in the figure to denote direct relationships between them.

Neuroinflammation and neuron-glia crosstalk is thought to play significant role in the selective vulnerability of the EC to degeneration during AD (Fig. 2). Glia cells, including astrocytes and microglia, provide neurons with structural and trophic support, and play important roles in maintaining homeostasis and ionic gradients, neuronal survival, clearing neurotransmitters of synapses, mediating immune responses, and reducing oxidative stress (Cameron and Landreth, 2010; Markiewicz and Lukomska, 2006; Matias et al., 2019; Ransom and Ransom, 2012; Zhang et al., 2010). Homeostatic perturbations due to neurochemical or physiological imbalances in neurons can cause astrocytes and microglia to release inflammatory molecules including cytokines in a bid to maintain cellular integrity and repair damaged cells. However, aberrant activation of astrocytes and microglia during chronic inflammation can result in neuronal death through the release of ROS and other nitrating molecules (Aloisi, 2001; Neumann, 2001; Simon et al., 2019).

Perturbations and neuroinflammatory changes involving both astrocytes and microglia have been well described in the EC of AD brains, where glia dysregulation and proinflammatory molecules play a central role in degeneration during AD (Asai et al., 2015; Criscuolo et al., 2017; Janelsins et al., 2005; Thangavel et al., 2013). For example, the EC of AD brains shows increased immunoexpression of the proinflammatory molecule glia maturation factor (GMF), glia fibrillary acidic protein (GFAP) labelled reactive astrocytes, ionized calcium-binding adaptor molecule-1 and activated microglia, and these changes are more concentrated at sites with amyloid plaques and NFTs (Thangavel et al., 2013). Changes in glia can be driven both by Aβ and the response to neuronal injury, but Aβ can also dysregulate the development of glial function in the EC. Yeh et al. (2011) analyzed astroglia morphology in the EC through surface and volume measurements of GFAP profiles in a triple transgenic mouse model of AD which harbours APPswe, PS1M146V and TauP301L mutations (3xTg-AD), and found that both the surface and volume of astroglia were reduced as compared to controls at a very early age (1 month). The reductions were maintained, and interestingly, it was found that the appearance of Aβ deposition at 12 months did not trigger astroglia hypertrophy. This susceptibility of entorhinal astrocytes to dysfunction triggered by amyloid beta during development suggests multiple potential mechanisms through which Aβ may promote dysfunction of glia, and contribute to the early vulnerability of EC to degeneration during the progression of AD.

Intrinsic differences between astrocytes in distinct brain regions, as reflected in heterogeneity in the expression profile of proteins involved in astrocyte morphology and function (Chai et al., 2017; Middeldorp and Hol, 2011; Waller et al., 2016), is likely to contribute to the selective vulnerability of brain regions such as the EC to age-related cognitive decline and AD (Matias et al., 2019). The EC shows greater numbers of glutamine synthetase (GS) labeled astrocytes as compared to GFAP containing astrocytes (Anlauf and Derouiche, 2013), while hippocampal astrocytes are widely stained for GFAP (Bushong et al., 2002; Chai et al., 2017). Astrocytic glutamate transporters are well known to remove glutamate from excitatory synapses and to prevent excitotoxicity due to excessive accumulation of glutamate. Further, GS converts glutamate to glutamine, and dysregulation of the glutamate-glutamine cycle can contribute to excess glutamate, excitotoxicity and neuronal loss in AD (Walton and Dodd, 2007). The prevalence of GS-type astrocytes in the EC (Anlauf and Derouiche, 2013) is consistent with the importance of this mechanism for regulating excitatory transmission among the large population of principal neurons, and the dysfunction of GS astrocytes may contribute to the susceptibility of the EC early in AD.

Neuroinflammation mediated by microglia may also contribute to the early vulnerability of the EC during AD. In the EC, but not the hippocampus, 3xTg-AD mice show the early upregulation of tumor necrotic factor (TNF)-alpha and monocyte chemoattractant protein (MCP-1), and these changes are correlated with increases in F4/80-positive microglia and macrophages (Janelsins et al., 2005). F4/80 protein is the murine homolog of the EGF-like module-containing mucin-like hormone receptor-like 1 that has been extensively used as a marker for mouse macrophage populations (Austyn and Gordon, 1981). Aβ can also activate microglial receptors, and trigger advanced glycation end products that may contribute directly to EC vulnerability and progression of synaptic and behavioral deficits (Criscuolo et al., 2017). Taken together, the uniqueness in EC glia protein expression patterns, as well as selective dysfunction of astrocyte and microglia coupled with neuroinflammation may account for early degeneration of EC neurons during AD-type pathogenesis. There is a lack of specific glia regional markers to fully identify the unique glia morphologic and functional properties, but the development of such markers is expected to allow investigations to better determine the roles of glia in the early vulnerability of the EC.

## Amyloid or tau-mediated activation of cell death kinases the EC during AD

Neuronal death during AD has been attributed to both Aβ and tau dysregulation as shown in Fig. 2 (Desikan et al., 2012; Lue et al., 1999; Näslund et al., 2000; Sipos et al., 2007). The original amyloid cascade hypothesis proposed that dysmetabolism of amyloid precursor protein (APP) causes an increase in amyloid plaque deposition and a pathological cascade resulting in NFT formation and neuronal death (Hardy and Allsop, 1991). More recent findings have found that soluble oligomeric Aβ, rather than insoluble amyloid plaques, have a more potent role in initiating cell death in AD (Barghorn et al., 2005; Christensen et al., 2008; Demuro et al., 2010; Gong et al., 2003; Lacor et al., 2007). Tau can also cause synaptic dysfunction independently of Aβ (Spires-Jones and Hyman, 2014), and may contribute to neuronal death through glutamate excitotoxicity (Amadoro et al., 2006). Interestingly, it has also been shown that tau is an essential modulator of Aβ-induced neuronal death during AD (Reifert et al., 2011; Song et al., 2008; Takashima et al., 1993; Zheng et al., 2002).

The loss of neurons in AD occurs very early in the EC (Gómez-Isla et al., 1996), and several reports indicate that aberrant cell cycle activation in postmitotic neurons occurs during early neuronal degeneration in AD (Busser et al., 1998; Hoozemans et al., 2002; Nagy et al., 1997; Vincent et al., 1997; Yang et al., 2006). Further, EC neurons appear to be particularly susceptible to mechanisms of cell death and apoptosis early in AD (Yang et al., 2006). The EC has a higher neuronal atrophy rate as compared to the hippocampus in AD subjects (Du et al., 2004), and interestingly, the density of cell-cycle-positive neurons in the EC is greater in subjects with mild cognitive impairment than in post-mortem AD brains (Yang et al., 2003). Also, the EC appears to be susceptible to cell-cycle events as kainic-acid-induced neuronal death in the hippocampus was followed by upregulation of several cell-cycle proteins of the G1, G2 and S phases in the EC (Hernández-Ortega et al., 2007). Intriguingly, the increased expression of these cell-cycle proteins in the EC is accompanied by progressive expression of proteins related to AD including Tau phos ser396/Ser404 and APP (Hernández-Ortega et al., 2007). Thus, the EC neurons may be particularly susceptible to cell-death stimuli early during the progression of AD.

Although the field is still nascent, evidences of the molecular mechanisms driving selective activation of cell-death pathways in the EC during AD are beginning to emerge. The selective activation of stress and cell-death kinases by Aβ or/and tau may contribute to the vulnerability of the EC to neuron loss (Fig. 2). There is increasing evidence that the cell stress-kinase p38 mitogen activated protein kinase (MAPK) plays an important role in AD pathophysiology (Munoz and Ammit, 2010). Aβ rapidly induces phosphorylation of p38 MAPK in the EC (Criscuolo et al., 2015), and activation of p38 MAPK can mediate early apoptosis by phosphorylating intracellular enzymes and cellular proteins involved in apoptosis (Kawasaki et al., 1997; Qi et al., 2004). This is supported by findings in the J20 mice (a transgenic model overexpressing human APP with familial AD mutations), that reducing the Aβ-mediated activation of stress-related kinases p38 including MAPK, and c-Jun N-terminal kinase (JNK), inhibits early synaptic dysfunction in layer II of the EC (Criscuolo et al., 2017). Similarly, specific MAPK pathways are activated in the EC about 3 to 6 h following systemic administration of kainic acid in rats, as shown by phosphorylated JNK and phosphorylated p38 immunoreactivity, phosphorylated c-Myc expression, decreased phosphorylated CREB immunoreactivity and strong phosphorylated c-Jun immunoreactivity. These kinases, which commit cells to apoptosis, may be the molecular basis for the vulnerability of the EC to cell death during the onset and progression of AD (Ferrer et al., 2002). This body of evidence supports a central role for cell-death kinases and cell-cycle dysregulation in neuronal death observed early in the EC during AD. These observations could have important implications for understanding of the molecular basis of the selective vulnerability of the EC during the onset and progression of AD.

## Conclusions

Though the full mechanistic spectrum accounting for the unique response of the EC to intrinsic and extrinsic neurotoxic stimuli leading to AD has not been fully determined, we have discussed the emerging understanding of the biochemical and anatomical variabilities that are specific to this brain region which may underlie molecular and functional neurodegeneration in AD. Studies reviewed here indicate that the EC may show selective vulnerability during the early pathogenesis of AD due to interacting factors including genetic and physiological differences in neuronal subpopulations, the expression level of neurochemicals modulating neural functions, and the sensitivity of multiple intracellular signalling cascades to triggers of AD pathology including Aβ protein.
